# Naturalisation and Immigrant Earnings: Why and to Whom Citizenship Matters

**DOI:** 10.1007/s10680-019-09540-1

**Published:** 2019-10-18

**Authors:** Floris Peters, Hans Schmeets, Maarten Vink

**Affiliations:** 1 Department of Political Science, Maastricht University, Grote Gracht 90-92, 6211 SZ Maastricht, The Netherlands; 2 Statistics Netherlands, CBS-weg 11, 6412 EX Heerlen, The Netherlands

**Keywords:** Citizenship, Immigration, Labour market, Netherlands

## Abstract

The notion that naturalisation matters for the economic integration of immigrants is well established in the literature, but why and to whom that is, remains surprisingly ambiguous. The citizenship premium is traditionally assumed to result from increased labour market access and positive signalling towards employers, but these mechanisms fail to explain increased earnings derived from paid employment, which has been the predominant focus in most studies. We argue that naturalisation needs to be understood in the context of the life course, as immigrants anticipate rewards and opportunities of citizenship acquisition by investing in their human capital development. Insofar as naturalisation subsequently leads to higher earnings, we expect that the citizenship premium mostly reflects better employment opportunities rather than access to better paying jobs. To test these assumptions, we use high-quality register data from Statistics Netherlands, covering the period 1999–2011. These data contain almost all registered foreign-born individuals in The Netherlands (*N* = 74,531) and allow us to track immigrant cohorts over time. Results show that naturalisation confers a one-time boost in earnings after naturalisation, but particularly for migrants from economically less developed countries and unemployed migrants. Furthermore, earnings develop faster leading up to naturalisation than afterwards, consistent with the notion of anticipation. The relevance of citizenship for employed immigrants in part results from an increase in working hours, but is not explained by variation in labour market sectors. We conclude that citizenship matters in terms of earnings from labour, but that its impact is not universal and manifests predominantly leading up to naturalisation.

## Introduction

Foreign-born individuals hold a weaker position in the labour markets of Western countries than natives (Heath and Cheung 2007; OECD 2015). This disadvantaged position can be attributed to discrepancies in relevant human capital endowment (Friedberg 2000), statistical discrimination (Arrow 1972; Dustmann 2000), and differing incentives to invest in one’s labour market potential (Chiswick and Miller 2001; Dustmann 2000). The (economic) incorporation of immigrants is high on the agenda of policy makers in OECD countries, and there is substantial political and academic interest in instruments or policy which may increase the probability of settlement success. One of the potentially promising vehicles to facilitate the process of immigrant integration is citizenship acquisition (OECD 2011). This paper analyses the relationship between naturalisation and labour market integration, specifically focusing on income from labour.

The relevance of citizenship acquisition for the labour market integration of immigrants—particularly in terms of earnings—has been a growing research subject over the last decades [see Helgertz et al. (2014, p. 343) and Gathmann (2015) for an overview]. Although citizenship acquisition is traditionally assumed to positively affect immigrant earnings, empirical findings do not consistently support this notion (Bevelander and Pendakur 2012; Bevelander and Veenman 2008; Bratsberg and Raaum 2011; Bratsberg et al. 2002; Chiswick 1978; Engdahl 2011; Steinhardt 2012). The relationship between citizenship and economic integration is complex, and the mixed evidence for a citizenship premium is often attributed to the methodological challenge of establishing a causal link between naturalisation and positive labour market outcomes (Helgertz et al. 2014, p. 338). More specifically, individuals who naturalise may differ from those who do not in terms of characteristics such as motivation or ability, which are hard to measure and control for, thus introducing the risk of overestimating the relevance of citizenship (Bratsberg et al. 2002; Helgertz et al. 2014; Steinhardt 2012). However, even when accounting for the so-called self-selection bias using panel data, the contradictory findings persist. Remarkably, this ambiguity is often taken as a challenge to the overall relevance of citizenship for integration outcomes of immigrants. This may be related to the fact that most studies in this field of literature are devoted to the question whether a citizenship premium exists or not. However, the universal effect this binary approach implies seems unlikely. Migrants have different motivations to naturalise, and the legal and financial obstacles to naturalisation differ between host countries, change over time, and may not be equally relevant to all migrant groups. An alternative explanation for the heterogeneous results may thus be that citizenship matters only for particular migrant groups, in certain countries, or through specific pathways to citizenship. Testing those assumptions requires a better understanding of the mechanisms underlying the citizenship premium. Hence, the important question in our view is not so much whether a citizenship premium exists or not, but particularly why and for whom this is the case.

This paper contributes to that field of literature by addressing this question in two ways. First, we develop the existing theoretical framework further and explicitly reflect on the determinants and mechanisms underlying the citizenship premium. Second, we build on the traditional methodological strategy of Bratsberg et al. (2002) by performing distributed fixed-effects analyses, which provide more detailed information on the temporal dynamic between citizenship acquisition and labour market outcomes. These two innovations shed new light on the question to whom and why naturalisation matters, respectively. We make use of Dutch register data from Statistics Netherlands. This individual-level dataset is based on municipal population registers and complemented by information from the Dutch System of Social Statistical Datasets. These data enable us to track the citizenship status, labour market performance, and other relevant socio-economic and demographic characteristics of almost all registered first-generation immigrants who migrated to The Netherlands between 1999 and 2002 for a period of 10 years (*N* = 74,531).

The paper is structured as follows: first, we briefly outline the Dutch context in terms of the migrant population and citizenship policies. Subsequently, the state-of-the-art on citizenship and labour market integration is discussed, and we reflect on the traditional mechanisms and theoretical assumptions in the literature. We then add to the existing theoretical framework, arguing that citizenship acquisition requires investment in relevant skills and knowledge leading up to naturalisation and that this ‘anticipation effect’ should be apparent in the labour market performance prior to citizenship acquisition (Bratsberg et al. 2002, p. 590; Peters et al. 2018). Next, the methodological approach and research design are described, followed by an overview and discussion of our empirical findings. Finally, we summarise our main results and discuss their implications.

## Context: Citizenship Policy and Labour Market Access in The Netherlands

In The Netherlands, the requirements for naturalisation are stipulated in the revised Dutch Nationality Act, introduced on April 1st, 2003. Migrants are eligible for citizenship acquisition when at least 18 years of age, having a residence permit for an undefined period of time and residing legally in The Netherlands for an uninterrupted period of 5 years. If an individual is the registered partner of a Dutch national for three consecutive years, he or she is exempted from the normal residence requirement, in which case only a non-temporary residence permit and principal residence in The Netherlands is required. Furthermore, migrants should not constitute a danger to public order (i.e. have no criminal record). In principle, dual citizenship is not allowed in The Netherlands, although there are many exceptions to the renunciation requirement. Exceptions include being the registered partner of a Dutch national or when renunciation of the original nationality is not legally possible or cannot be reasonably demanded. Finally, migrants who wish to naturalise should pass the language and integration requirement by successfully completing a formalised naturalisation test. Migrants are required to read, write and speak Dutch at level A2 of the Common European Framework of Reference for Languages, and should possess sufficient knowledge of the Dutch society. The Dutch nationality guarantees a secure legal status in The Netherlands, as well as full voting rights. Individuals without the Dutch nationality, but who originate from the European Economic Area (EEA), have unrestricted access to the labour market, with the exception of a small number of professions that are reserved for Dutch citizens, namely jobs in the army and high-ranking positions in law and the public sector. Naturalisation provides access to those jobs. Foreign individuals who originate from outside the EEA either need a work permit or the employer needs to have permission to hire an employee from outside the EEA, who in that case only needs a residence permit.

## Theoretical Framework

Literature on the determinants of economic integration of immigrants generally draws on the concept of human capital (Becker 1964). In the framework of human capital theory, opportunities and success of individuals in the labour market depend on their resources and skills. Resources include social networks and relevant labour market information, while skills refer to for instance educational qualifications, training and work experience. First-generation immigrants face structural disadvantages in the labour market compared to natives due to the diminished relevance or recognition of their original human capital in the host country (Algan et al. 2010; Friedberg 2000). For instance, migrants often have a limited mastery of the host country language compared to natives (Chiswick and Miller 2001; van Tubergen and Kalmijn 2005). Furthermore, the social capital of immigrants is often less effective at facilitating upward mobility due to the ethnic composition of the network (Lancee 2010). Moreover, employers may favour a native job candidate in light of the perceived risk of short-term emigration (Dustmann 2000) or because of statistical discrimination (Arrow 1972).

Does citizenship acquisition have the potential to level the playing field? Traditionally, the literature points to three mechanisms that explain why naturalisation might mitigate some of the above disadvantages and promote economic integration (Bratsberg et al. 2002, p. 569; OECD 2011). First, naturalisation removes restrictions on occupations that are reserved for citizens, such as jobs in the public sector, law and military. Second, employers do not have to pay the administrative costs associated with the verification of work permits when hiring a naturalised migrant. Third, in the context of statistical discrimination, citizenship may function as a positive signalling device. Employers may assume that the naturalised status of a migrant is indicative of positive selection, reducing the risk of hiring said individual.

These mechanisms imply a positive effect of citizenship on the labour market performance of immigrants. However, an examination of empirical findings in the literature reveals substantial ambiguity. Whereas some studies identify the expected positive relationship (Bevelander and Pendakur 2012; Bratsberg et al. 2002; Gathmann and Keller 2017; Steinhardt 2012), others find no such relationship, or even a negative association (Bratsberg and Raaum 2011; Chiswick 1978; Engdahl 2011). Traditional studies in this field of literature are typically based on cross-sectional data,^1^ which is frequently criticised for its inability to analyse the causal nature of the relationship. Citizenship acquisition is an inherently selective process. As such, individuals who naturalise may be positively selected with regard to their labour market potential. Although empirical findings based on panel data consistently confirm this hypothesis (Bratsberg et al. 2002; Helgertz et al. 2014; Steinhardt 2012), controlling for selection into naturalisation does not fully explain the contradictory findings in the literature. This enduring empirical ambiguity is often seen as a challenge to the existence of a citizenship premium in general. Alternatively, the ambiguity may invite us to theorise on its determinants, an approach which is remarkably absent in the literature. To address this gap, we first reflect on why the traditional mechanisms outlined above fail to explain empirical findings and subsequently introduce complementary mechanisms which may facilitate a better understanding of why naturalisation matters for some migrants under certain conditions, and not for others.

### Why Does Citizenship Acquisition Matter?

The literature on the citizenship premium predominantly analyses immigrant earnings, occasionally complemented by additional analyses on having employment or not. Regardless of the operationalisation of economic integration, the theoretical framework remains the same, focussing on the three established mechanisms: increased labour market access, reduced administrative costs and positive signalling. However, these mechanisms are not necessarily equally relevant in terms of having employment and earnings from labour. A focus on immigrant earnings implies a different research population than an analysis of employment. Indeed, migrants with earnings from labour per definition have employment. Arguably, being employed serves as a positive signalling device in its own right. These migrants therefore do not need the host country citizenship as much as migrants who are unemployed. Hence, there seems to be a mismatch between the most common operationalisation of economic integration in the literature, namely earnings, and one of the most important mechanisms, namely positive signalling, which arguably is more relevant in the context of having employment. As this paper focuses on income from labour, we generally expect to observe little to no one-time boost after naturalisation.

Traditionally, the relevance of citizenship acquisition in the labour market is almost exclusively attributed to the risk calculation of employers. Individuals who naturalise are largely considered passive beneficiaries of the positive impact of citizenship. As such, the effect of naturalisation is generally understood as a dichotomous before-after phenomenon (e.g. Helgertz et al. 2014, p. 351). Indeed, the notion of positive signalling, reduced administrative costs and increased access to the labour market only applies to migrants who have successfully naturalised. But citizenship acquisition is not an abrupt legal status transition, but rather a process that requires careful planning and preparation leading up to naturalisation. Although there is significant cross-national variation in citizenship policies (Vink and de Groot 2010), most European countries have formalised the conditions for eligibility into not only a minimum period of (legal) residence, but also obligatory language and civic integration requirements. These conditions imply that migrants need to invest in relevant skills and knowledge, most notably linguistic capabilities, if they wish to naturalise in the future. Moreover, from a life course perspective, migrants who have decided to naturalise have a strong incentive to invest in such recourses, since their future is likely focused on long-term residence in the host country. This human capital development in anticipation of citizenship acquisition may increase labour market opportunities already prior to naturalisation. While such an anticipation effect precedes the legal status transition, and would stem from investment in for instance language skills, it can still be considered a citizenship effect because it is directly linked to the intention to naturalise. In other words, the accelerated accumulation of host country-specific human capital is predicated on naturalisation in the future and would not have happened in the counterfactual where the migrant did not naturalise.

The notion of an anticipation effect has already been coined more than a decade ago by Bratsberg et al. (2002, p. 590)^2^ but surprisingly, has not received much attention in the state-of-the-art literature. This may be related to the fact that any positive labour market outcomes prior to naturalisation are typically labelled as selection (Helgertz et al. 2014, p. 344). Such effects are therefore considered bias that should be isolated, rather than an interesting phenomenon that needs to be studied. We argue, however, that it is important to identify the mechanisms underlying the positive effects prior to naturalisation for two reasons.

First, the state-of-the-art empirical strategy (Bratsberg et al. 2002) is specifically designed to capture selection resulting from time-invariant characteristics (such as cognitive ability). It is, however, less able to measure (and control for) anticipation, since this manifests specifically surrounding the moment of naturalisation. Adequately controlling for the positive effects prior to naturalisation thus requires deeper insight in where these effects are coming from.

Second, a better understanding of the effects prior to naturalisation may play an important role in theorising to whom and under which conditions citizenship matters. Several studies have shown that citizenship acquisition has a stronger effect on labour market outcomes if it is acquired relatively early in the settlement process (Gathmann and Keller 2017; Peters et al. 2018). This finding is difficult to explain through the more traditional mechanism of positive signalling, but may be rationalised by the notion of anticipation. Since migrants gradually accumulate host country-specific human capital over time, accelerated investment in these skills becomes less relevant the longer migrants reside in the host country. Investing in for instance language capabilities is more likely to matter after 4 years of residence than after 10 years. In other words, the mechanism of anticipation may explain why the relevance of citizenship is conditioned by the speed of naturalisation. In contrast, if the positive effects prior to naturalisation are solely due to for instance cognitive ability or motivation, then the process by which citizenship is acquired should not matter. An analysis of the anticipation mechanism may thus reveal whether citizenship matters predominantly through selection and positive signalling, or whether it can also incentivise integration on the part of migrants. Our hypothesis is as follows:

#### **H1**

The income from labour of immigrants who naturalise develops faster prior to the moment of naturalisation than afterwards.

### To Whom Does Citizenship Matter?

Insofar as citizenship acquisition matters, does it matter equally to all migrant groups? The literature seems to acknowledge that this is unlikely, since most studies perform separate analyses for migrants from various (parts of) continents (Bratsberg and Raaum 2011; Engdahl 2011, 2014; Helgertz et al. 2014; Steinhardt 2012). While we agree with the intuition that the relevance of citizenship for labour market outcomes is likely conditioned by the origin context, the literature provides little to no guidance as to why that would be the case. Such mechanisms are difficult to identify based on analyses stratified by origin regions due to substantial internal variation within those regions. As such, heterogeneity in the effect of naturalisation is observed, but not explained. Analyses focusing on specific characteristics of origin countries may facilitate a better understanding of how the origin context interacts with the mechanisms underlying the citizenship premium.

The signalling potential of citizenship is assumed to promote labour market integration by positively affecting the risk calculation of employers. However, employers will associate the hiring of individuals from certain migrant groups with higher risk than others. Migrants who are assumed to be negatively selected by employers with regard to their productivity and general labour market performance arguably stand to benefit from naturalisation most, as citizenship acquisition has the potential to mitigate the negative consequences of statistical discrimination for these migrants. This raises the question which migrant groups hold a more negative reputation in the labour market. Some research suggests that the citizenship premium is stronger for migrants for whom the probability of having paid employment is lowest, such as those from economically less developed countries of origin (Bratsberg et al. 2002, p. 590; Fougère and Safi 2011, p. 138). Our expectation is as follows:

#### **H2**

The positive effect of citizenship acquisition on income from labour is stronger for immigrants from economically less developed countries of origin.

## Data and Methodology

We make use of register data from Statistics Netherlands to analyse the potential relationship between citizenship acquisition and earnings from labour. The data are derived from the Dutch System of Social Statistical Datasets (Bakker et al. 2014). This micro-level dataset allows us to track relevant characteristics of foreign-born individuals over time. More specifically, we observe individual characteristics every 6 months, and country characteristics with yearly precision, over the period of 1999 until 2011. Individuals are followed from the moment of arrival in The Netherlands, until they reach the end of the observation period (January 2012), or leave The Netherlands, with bi-annual observations (January 1 and July 1). Migrants are tracked from the moment of migration onwards because the hypothesised anticipation mechanism is expected to be relevant leading up to citizenship acquisition and thus prior to the moment of eligibility for naturalisation. We focus on migrants who arrived in The Netherlands between 1999 and 2002. The reason for this cohort selection is that micro-data on labour market characteristics is only available from 1999 onwards. Moreover, excluding cohorts after 2002 allows us to observe all cohorts for more than 9 years. The observation period for all cohorts is fixed at a maximum of 10 years of residence. We further restrict the sample to migrants who become eligible for naturalisation during the observation period. More specifically, we determine on the basis of the moment of arrival in the host country and the partner status over time when migrants would fulfil the residence requirement for naturalisation (normally after 5 years of residence, but potentially sooner if one is the registered partner of a Dutch national). If migrants drop out of the dataset before this point in time (typically because of outmigration), then these individuals are not included in the analyses. This ensures a cleaner comparison between, on the one hand, migrants who naturalised and, on the other hand, those who could have done so, but chose not to.^3^

The analysis focusses on first-generation immigrants, defined as foreign-born individuals of whom both parents were born abroad. Furthermore, we exclude migrants who acquired Dutch citizenship prior to arriving in The Netherlands, such as those born in Suriname before 1975 or in The Netherlands Antilles, who are often Dutch citizens by birth. As is common in labour market research, we perform separate analyses for men and women. Also, we exclude migrants who are inactive in the labour market, namely students, retirees and individuals with health problems or disabilities that impede their participation in the labour market. To further focus the selection on individuals with an equal incentive to integrate into the labour market, we restrict the sample to migrants aged between 20 and 50 years at the moment of arrival in The Netherlands (Engdahl 2014, p. 11; Helgertz et al. 2014, p. 347).

The dependent variable in this paper is income from labour, measured as the common logarithm of monthly wages, corrected for inflation based on the Consumer Price Index. This includes income derived from employment and self-employment. Our independent variables include naturalisation, age at the moment of migration, years since migration, the partner status, having children in the household, the mean working hours per year, the labour market sector, and the level of economic development and EU membership of the country of origin. Naturalisation is operationalised as possession of the Dutch citizenship. Note that the registers do not include information on the naturalisation process, and we are thus unable to distinguish between migrants who are interested in naturalisation but have not (yet) met the formal requirements and those who do not wish to naturalise. However, as detailed above, we exclude migrants who are able to acquire the Dutch citizenship through procedures other than naturalisation, such as option, acknowledgment or birth. We track the registered partner of immigrants over time, differentiating between migrants with a foreign-born foreign partner, a foreign-born Dutch partner (a partner who has naturalised) and a native partner. We classify migrants of whom the youngest child in the household is younger than 18 as having children. In terms of origin characteristics, we focus on economic development, based on the Human Development Index (UNDP 2014) and EU membership of origin countries. Due to the relatively small cohort selection, there is a strong relationship between years since migration and the years of observation. More detailed analyses confirm multicollinearity when the observation years are added to the models (VIF > 7). Controls for the observation years are therefore not included in the analyses.

To analyse the data, we make use of individual fixed-effects regression. This methodology is used to control for unmeasured, time-invariant heterogeneity within individuals. Basically, individual fixed-effects implies a control for each individual in the dataset. In practice, this means that we control for all characteristics that do not vary within the observation period, such as the age at migration, migration motive and country of origin, but also endogenous characteristics such as commitment, motivation and ability. This strategy can be considered the state-of-the-art methodology to isolate selection bias in this field of literature (Bratsberg and Raaum 2011; Bratsberg et al. 2002; Engdahl 2014; Helgertz et al. 2014; Steinhardt 2012).

We follow the empirical strategy developed by Bratsberg et al. (2002), by measuring the relevance of naturalisation through three parameters. The first parameter ($\alpha_{0}$) is a dummy measuring whether a migrant is naturalised or not ($N_{it}$), thus capturing a potential one-time shift in earnings from labour after naturalisation. The second parameter ($\alpha_{1}$) is an interaction between a time-invariant dummy measuring whether a migrant naturalises during the observation period and years since migration ($D_{i} X_{it}$), which captures the differentiated effect of years since migration for migrants who naturalise, and those who do not. This parameter thus captures a potentially steeper slope for migrants who naturalise, including prior to naturalisation. The third parameter ($\alpha_{2}$) measures a gradual change in earnings from labour after naturalisation. This is an interaction between a dummy that is set to unity when a migrant has naturalised at a given observation $\left( {N_{it} } \right)$ and years since naturalisation, measured as years since migration $(X_{it} )$ minus the year of naturaliation $\left( {X_{iN} } \right)$. Years since naturalisation is thus a continuous variable that is negative prior to naturalisation, positive after naturalisation and zero in the year of citizenship acquisition. A positive coefficient indicates a more positive development of income from labour after naturalisation, whereas a negative coefficient means that income develops faster among migrants who are not (yet) naturalised. All models include an additional vector of control variables ($Z_{it}$), as well as individual fixed-effects ($u_{i}$). The main econometric equation is as follows:

1 $$\ln \left( {Y_{it} } \right) = \alpha_{0} N_{it} + \alpha_{1} D_{i} X_{it} + \alpha_{2} N_{it} \left( {X_{it} - X_{iN} } \right) + \gamma X_{it} + \delta Z_{it} + u_{i} + \varepsilon_{t}$$

A notable shortcoming of models based on Eq. (1) is that it is difficult to identify how exactly the earnings profile of naturalising migrants develops surrounding the moment of citizenship acquisition. Although parameters $\alpha_{1}$ and $\alpha_{2}$ provide information on earnings from labour before and after naturalisation, they are particularly suited to analyse average constant effects associated with for instance high levels of motivation or ability. However, if anticipation is one of the driving factors behind the steeper earnings profile prior to naturalisation (as theorised in this paper), then we would expect the positive effect to peak around the moment of naturalisation. A positive coefficient for parameter $\alpha_{1}$ and a negative coefficient for parameter $\alpha_{2}$ would be consistent with this notion, but could also imply that the positive effect continues after naturalisation, but at a reduced rate compared to the period before citizenship acquisition. Note that the expected peak around the moment of naturalisation is not to say that migrants will not continue to gradually accumulate host country-specific human capital, but rather that the unique effect of citizenship will decrease as the anticipatory incentive disappears.

To analyse the temporal development of earnings from labour in more detail, we follow the approach of Engdahl (2014, p. 17) by performing a distributed individual fixed-effects regression. More specifically, parameter $\alpha_{3}$ is next to a categorical variable that introduces nine separate dummies for the following periods: more than 3 years prior to naturalisation, each of the 3 years leading up to naturalisation, the year of naturalisation, each of the first 3 years after naturalisation, and a final dummy for all subsequent years. Since this method is specifically designed to identify how rather than whether citizenship matters, we focus only on migrants who naturalise during the observation period ($j$). The econometric equation is as follows:

2 $$\ln (Y_{jt} ) = \mathop \sum \limits_{b = - 3}^{3} \alpha_{3} X_{jN + b} + \gamma X_{jt} + \delta Z_{jt} + u_{j} + \varepsilon_{t}$$

In contrast to models based on Eq. (1), where we only capture the average constant effect of naturalisation, this less restrictive model provides a more detailed account of how earnings from labour develop during the period before and after naturalisation. With migrants in the period more than 3 years prior to naturalisation as the reference category, this variable details relative changes in income from labour in the period surrounding citizenship acquisition and allows us to confirm whether these changes in the earnings profile are consistent with the hypothesised anticipation mechanism.

Table 8 (see the Appendix of the paper) contains descriptive statistics on the Log labour income of male and female immigrants with employment.^4^ As expected, male immigrants enjoy higher earnings than female immigrants. Furthermore, the income from labour of migrants who naturalise gradually increases surrounding the moment of naturalisation, although it never reaches the average level of immigrants who do not naturalise. This comparatively lower earnings profile is likely the result of characteristics that are associated with immigrants’ propensity to naturalise. Migrants who are most interested in naturalisation, such as those from economically less develop countries of origin, also tend to enjoy lower earnings from labour. The relevance of the additional personal and contextual characteristics corresponds to our expectations and is in line with earlier studies on labour market integration of immigrants^5^ (Jasso and Rosenzweig 1986; Kanas et al. 2011; Kogan 2011; Lancee 2010; van Tubergen et al. 2004).

## Analysis

Table 1 provides the results of the three parameters on naturalisation detailed in Eq. (1), as well as a number of control variables which feature substantial changes over time, and are thus not captured by the individual fixed-effects. Model 1 contains only observations of individuals with employment, whereas Model 2 also includes observations where migrants are unemployed, which is of interest in light of our argument that the traditional mechanisms underlying the citizenship premium are particularly relevant to get access to the labour market rather than earnings from labour. Results show that men and women enjoy a minor one-time boost in Log labour income of 1.8% (10^0.008^) and 1.4% (10^0.006^) after naturalisation. These findings provide some support for the signalling potential of citizenship and are consistent with findings from earlier studies in Norway (Bratsberg and Raaum 2011, p. 197), Sweden and Denmark (Helgertz et al. 2014, p. 352), Germany (Steinhardt 2012, p. 818) and the USA (Bratsberg et al. 2002, p. 582). Yet, these studies provide only limited theoretical guidelines that explain why we only observe a relatively modest effect. Our interpretation is that the signalling potential of citizenship is particularly relevant for unemployed migrants, since having employment serves as a positive signalling device in its own right. The interaction between whether a migrant naturalises during the observation period and years since migration is positive and statistically significant. This confirms that migrants who naturalise perform better in the labour market, including prior to naturalisation. The coefficient of the interaction between whether an individual has naturalised and years since naturalisation is negative, indicating that the earnings profile develops faster for migrants who are not (yet) naturalised. In sum, income from labour particularly increases leading up to naturalisation. These findings are contrary to the traditional understanding of a citizenship premium, but consistent with the notion of anticipation. Migrants anticipate potential rewards and opportunities of naturalisation by investing in their own human capital development. In line with earlier theorisation in the literature (Bratsberg et al. 2002, p. 582–583), we hypothesise that the steeper wage gains prior to naturalisation are the result of these investments.

**Table 1:** Individual fixed-effects regression on (CPI-adjusted) Log labour income of male and female immigrants with and without paid employment, cohorts 1999–2002. *Source*: Statistics Netherlands

	Model 1				Model 2			
	Men		Women		Men		Women	
	Coef.	SE	Coef.	SE	Coef.	SE	Coef.	SE
Naturalisation								
Yes	0.008	0.002***	0.006	0.002**	0.092	0.009***	0.094	0.009***
No	Ref.	Ref.	Ref.	Ref.	Ref.	Ref.	Ref.	Ref.
Years since migration*naturalisation during observation period								
	0.022	0.000***	0.021	0.001***	0.155	0.001***	0.127	0.002***
Years since naturalisation*naturalisation								
	− 0.019	0.001***	− 0.013	0.001***	− 0.115	0.003***	− 0.083	0.003***
Years since migration								
	0.020	0.000***	0.022	0.000***	0.052	0.001***	0.075	0.001***
Partner								
No partner	Ref.	Ref.	Ref.	Ref.	Ref.	Ref.	Ref.	Ref.
Foreign-born foreign partner	0.017	0.001***	− 0.001	0.002	0.099	0.005***	0.021	0.006***
Foreign-born Dutch partner	0.016	0.002***	− 0.027	0.003***	0.296	0.007***	0.093	0.008***
Native-born Dutch partner	0.020	0.002***	0.006	0.002***	0.246	0.007***	0.183	0.006***
Children < 18 in the household								
Yes	0.009	0.001***	− 0.077	0.001***	0.035	0.005***	− 0.368	0.005***
No	Ref.	Ref.	Ref.	Ref.	Ref.	Ref.	Ref.	Ref.
	*N* = 40,204 Observations = 502,962 − 2 Log-likelihood = 945,707		*N* = 34,327 Observations = 389,668 − 2 Log-likelihood = 720,244		*N* = 40,204 Observations = 727,522 − 2 Log-likelihood = 2,434,748		*N* = 34,327 Observations = 614,459 − 2 Log-likelihood = 2,199,116	

Model 1 is restricted to observations of individuals with paid employment. Model 2 includes observations in which individuals are unemployed

**p < 0.01

***p < 0.001

Regarding our control variables, we observe a positive relationship between years since migration and earnings, all else constant. Furthermore, we find that having a partner is generally associated with increased earnings among men, whereas for women the effects are less positive (Kanas et al. 2011, p. 113). Having young children in the household has a modest positive effect on the Log labour earnings of men, whereas the effect is strongly negative for female immigrants. Clearly, these events have different implications in the life course of men and women, respectively.

In line with our expectations, citizenship acquisition only provides a limited one-time boost in earnings for employed men and women. However, if our expectation that the signalling potential of citizenship matters predominantly for employment rather than earnings holds, then the coefficient for the ‘naturalisation’ parameter should be more positive when migrants without employment are added to the model. Model 2 of Table 1 provides the results of the individual fixed-effects regression including observations where migrants are unemployed. Compared to the findings in Model 1, the coefficients are substantially larger.^6^ This suggests that the subsequent effect of naturalisation on immigrant earnings is stronger for migrants without employment. We theorise that this is the case because being employed has a positive signalling effect in its own right. Thus, these employed individuals do not need citizenship acquisition as much in the labour market. Moreover, for citizenship to have an effect for employed migrants implies the assumption that these migrants reorient themselves in the labour market after naturalisation. Our findings suggest that the traditional arguments for a citizenship premium are particularly relevant in the context of gaining access to the labour market (i.e. having employment) rather than occupational mobility (i.e. earnings).

Although the positive labour market outcomes prior to citizenship acquisition suggest an anticipation effect, they do not provide any indication regarding the exact shape of the slope before and after naturalisation. If anticipation is the underlying mechanism, then we would expect earnings from labour to peak around the moment of citizenship acquisition. To analyse this in detail, we perform a distributed individual fixed-effects regression based on Eq. (2). We analyse immigrants who naturalise during the observation period, since the focus of these analyses is not so much on whether citizenship matters or not (which is the purpose of Table 1), but rather how it matters. Table 2 shows that, as expected, immigrant earnings increase leading up to citizenship acquisition, and peak around the moment of naturalisation (the year before and after naturalisation for men and women, respectively). This confirms hypothesis 1 and is in line with our theorisation that the explicit decision to naturalise in the future results in the corresponding decision to invest more heavily in host country-specific human capital already prior to naturalisation (Bratsberg et al. 2002, p. 582). We assume that the more positive labour market performance prior to naturalisation is a reflection of said investment. After naturalisation, the additive effect of naturalisation decreases, particularly for male immigrants.^7^

**Table 2:** Distributed individual fixed-effects regression on (CPI-adjusted) Log labour income of male and female immigrants with paid employment who naturalise during the observation period, cohorts 1999–2002. *Source*: Statistics Netherlands

	Men				Women			
	Coef.	SE	95% conf. interval		Coef.	SE	95% conf. interval	
Naturalisation								
> 3 years prior to naturalisation	Ref.	Ref.			Ref.	Ref.		
3 years prior to naturalisation	0.025	0.003***	0.019	0.031	0.019	0.003***	0.013	0.025
2 years prior to naturalisation	0.039	0.003***	0.033	0.045	0.041	0.003***	0.035	0.047
1 year prior to naturalisation	0.047	0.003***	0.041	0.053	0.048	0.004***	0.040	0.056
Year of naturalisation	0.046	0.004***	0.038	0.054	0.051	0.005***	0.041	0.061
1 year after naturalisation	0.043	0.005***	0.033	0.053	0.053	0.005***	0.043	0.063
2 years after naturalisation	0.038	0.005***	0.028	0.048	0.049	0.006***	0.037	0.061
3 years after naturalisation	0.025	0.006***	0.013	0.037	0.044	0.007***	0.030	0.058
> 3 years after naturalisation	0.007	0.007	− 0.007	0.021	0.039	0.008***	0.023	0.055
Years since migration								
	0.031	0.001***	0.029	0.033	0.032	0.001***	0.030	0.034
Partner								
No partner	Ref.	Ref.			Ref.	Ref.		
Foreign-born foreign partner	0.003	0.002	− 0.001	0.007	− 0.009	0.003**	− 0.015	− 0.003
Foreign-born Dutch partner	0.037	0.003***	0.031	0.043	− 0.012	0.004**	− 0.020	− 0.004
Native-born Dutch partner	0.029	0.004***	0.021	0.037	0.004	0.003	− 0.002	0.010
Children < 18 in the household								
Yes	− 0.002	0.002	− 0.006	0.002	− 0.077	0.002***	− 0.081	− 0.073
No	Ref.	Ref.			Ref.	Ref.		
	*N* = 13,685 Observations = 158,589 − 2 Log-likelihood = 281,406				*N* = 12,971 Observations = 132,626 − 2 Log-likelihood = 236,309			

**p < 0.01

***p < 0.001

The next question is to whom citizenship matters. Analogous to the state-of-the-art literature (Bratsberg and Raaum 2011; Engdahl 2014; Helgertz et al. 2014; Steinhardt 2012), we start by performing separate analyses for large origin regions. Tables 3 and 4 provide results for men and women, respectively. First, we observe that earnings of naturalising migrants from all origin groups develop faster over time, including prior to naturalisation. Furthermore, income from labour develops faster prior to naturalisation than afterwards for all migrant groups except women from the Middle-East, indicated by the negative interaction between whether an individual is naturalised and years since naturalisation. In line with the aggregate analyses, we observe a one-time boost in Log labour income after naturalisation for male immigrants from Africa, Asia and the Middle-East, and female immigrants from Asia, South America and the Middle-East. More specifically, male immigrants enjoy an increase between 3.8 and 4.9% in Log labour income, and female immigrants an increase between 3.5 and 6.7%. In contrast, the coefficient is negative for women from the EU.

**Table 3:** Individual fixed-effects regression on (CPI-adjusted) Log labour income of male immigrants with paid employment, by origin regions, cohorts 1999–2002. *Source*: Statistics Netherlands

	EU	Non-EU Europe, North America and Australia	South America	Africa	Asia	Middle-East
	Coef.	Coef.	Coef.	Coef.	Coef.	Coef.
Naturalisation						
Yes	− 0.005	− 0.014	0.003	0.021***	0.019**	0.016***
	(0.006)	(0.010)	(0.009)	(0.003)	(0.007)	(0.003)
No	Ref.	Ref.	Ref.	Ref.	Ref.	Ref.
Years since migration*naturalisation during observation period						
	0.024***	0.013***	0.018***	0.015***	0.015***	0.022***
	(0.001)	(0.002)	(0.002)	(0.001)	(0.001)	(0.001)
Years since naturalisation*naturalisation						
	− 0.010***	− 0.012***	− 0.017***	− 0.016***	− 0.021***	− 0.018***
	(0.002)	(0.003)	(0.003)	(0.001)	(0.002)	(0.001)
*N*	12,073	3039	1190	9294	2678	13,100
Observations	164,026	33,130	13,973	113,884	33,814	144,135
− 2 Log-likelihood	243,561	73,814	22,704	188,058	64,718	283,357

Standard errors in parentheses. Results include controls for years since migration, the partner status and having young children in the household

**p < 0.01

***p < 0.001

**Table 4:** Individual fixed-effects regression on (CPI-adjusted) Log labour income of female immigrants with paid employment, by origin regions, cohorts 1999–2002. *Source*: Statistics Netherlands

	EU	Non-EU Europe, North America and Australia	South America	Africa	Asia	Middle-East
	Coef.	Coef.	Coef.	Coef.	Coef.	Coef.
Naturalisation						
Yes	− 0.008*	0.009	0.015**	0.007	0.028***	0.023***
	(0.004)	(0.007)	(0.006)	(0.005)	(0.006)	(0.007)
No	Ref.	Ref.	Ref.	Ref.	Ref.	Ref.
Years since migration*naturalisation during observation period						
	0.028***	0.027***	0.004**	0.016***	0.013***	0.013***
	(0.001)	(0.002)	(0.002)	(0.001)	(0.001)	(0.002)
Years since naturalisation*naturalisation						
	− 0.016***	− 0.031***	− 0.005**	− 0.009***	− 0.017***	− 0.001
	(0.001)	(0.003)	(0.002)	(0.002)	(0.002)	(0.002)
*N*	14,267	4518	2820	4783	5417	4897
Observations	172,505	37,423	29,994	48,410	60,727	40,609
− 2 Log-likelihood	291,839	78,116	54,849	88,141	113,877	85,679

Standard errors in parentheses. Results include controls for years since migration, the partner status and having young children in the household

*p < 0.05

**p < 0.01

***p < 0.001

Due to substantial demographic and institutional variation between these large origin groups, it is hard to identify underlying mechanisms that explain these empirical differences. We hypothesise that these findings may partly reflect variation in economic development between origin countries. Insofar as citizenship matters in terms of earnings from labour, we expect that it will be particularly relevant to migrants from less developed countries. Table 10 provides results of separate analyses for migrants with employment from low and high developed countries. Individuals have been classified as originating from low or high developed countries based on the median human development score. Results indicate that naturalising migrants from all origin groups perform better in the labour market, including prior to naturalisation. Moreover, the earnings profile develops faster prior to naturalisation than afterwards. However, the one-time boost in earnings is positive and statistically significant for migrants from less developed countries of origin, whereas this is not the case for those from high developed countries. Among migrants from less developed countries, we observe an increase of 3.2 and 4.7% in Log labour income for male and female immigrants, respectively. These findings provide support for hypothesis 2, in which we argue that citizenship particularly matters for vulnerable migrant groups who struggle in the labour market (Bratsberg et al. 2002, p. 590; Fougère and Safi 2011, p. 138). Migrants who are assumed to be negatively selected by employers, such as those from economically less developed countries of origin, will particularly benefit from the signalling potential of the host country citizenship to mitigate their disadvantaged position. Conversely, migrants from more developed countries may not face the same preconceptions and accordingly do not need the host country citizenship to compensate.

In sum, we observe an effect of citizenship on immigrant earnings even when controlling for endogeneity, but (1) it does not apply to all migrant groups and (2) does not solely manifest as a consequence of naturalisation itself but also from the decision to naturalise in the future. However, our findings do not provide an indication where these effects in Log labour income are coming from. On the one hand, naturalisation may facilitate access to higher paying jobs, but on the other hand, the effect might also stem from more working hours. To analyse this in detail, we include a control for working hours to the main model. Data on working hours are only available for migrant cohorts 2001 onwards, so we perform the main analysis (without a control for working hours) for cohorts 2001–2002 to facilitate the comparison and to provide a clear indication as to the relevance of working hours to the model. Table 5 shows the findings for men and women, respectively. Results from the model without a control for working hours are similar to those in the main analysis (Table 1), except that the upward shift in income is more pronounced. However, when a control for working hours is added to the model, this ‘Naturalisation’ coefficient changes back to the magnitude of the main analysis. For women, the coefficient even becomes negative. More generally, all the coefficients on naturalisation decrease when controlling for working hours. These findings thus suggest that insofar as there is an effect of naturalisation, it is in part explained by working hours.

**Table 5:** Individual fixed-effects regression on (CPI-adjusted) Log labour income and (CPI-adjusted) Log labour income when controlling for working hours of male and female immigrants with paid employment, cohorts 1999–2002. *Source*: Statistics Netherlands

	Men				Women			
	No control for working hours		Control working hours		No control for working hours		Control working hours	
	Coef.	SE	Coef.	SE	Coef.	SE	Coef.	SE
Naturalisation								
Yes	0.025	0.003***	0.009	0.002***	0.014	0.003***	− 0.005	0.002*
No	Ref.	Ref.	Ref.	Ref.	Ref.	Ref.	Ref.	Ref.
Years since migration*naturalisation during observation period								
	0.017	0.001***	0.007	0.000***	0.020	0.001***	0.007	0.001***
Years since naturalisation*naturalisation								
	− 0.013	0.001***	− 0.001	0.000***	− 0.010	0.001***	− 0.000	0.001
	*N* = 20,272		*N* = 20,272		*N* = 17,578		*N* = 17,578	
	Observations = 241,173		Observations = 241,173		Observations = 191,061		Observations = 191,061	
	− 2 Log-likelihood = 421,294		− 2 Log-likelihood = 335,248		− 2 Log-likelihood = 326,996		− 2 Log-likelihood = 234,933	

Results include controls for years since migration, the partner status and having young children in the household

*p < 0.05

**p < 0.01

***p < 0.001

Naturalisation has the potential to stimulate earnings from labour for some immigrant groups. But to what extent is the relevance of citizenship explained by variation in labour market sectors that migrants are employed in? Tables 6 and 7 provide estimates for employed men and women, respectively, when labour market sectors are added to the main model. Since information on labour market sectors is unknown for a number of individuals, we also repeat the main analysis (without controlling for labour market sectors) for the population where information on sectors is available. Our findings reveal some discrepancies in Log labour income between sectors (see also Tables 11 and 12 in the Appendix). Detailed analyses indicate that heterogeneity in Log labour income between labour market sectors is largely explained by discrepancies in levels of education, which are mostly captured by the individual fixed-effects (the relevance of education is analysed in detail in the chapter ‘Robustness analyses’). The substantial number of immigrants working in jobs in business services, such as call centres, packaging and office cleaning, enjoy relatively low earnings. In contrast, earnings are high in information and communication (e.g. system administrators, technical support of companies, radio and television) and the financial services (e.g. banks, investment companies, credit unions), although the number of migrants working in these sectors is comparatively small. Our data do not allow for a disentanglement of jobs in the public sector and the care sector. While a substantial proportion of immigrants are represented in this category (particularly female immigrants), earnings tend to be relatively low in the care sector, which may explain why the coefficients for this category are not higher. With regard to citizenship acquisition, when labour market sectors are added to the model, the coefficients of the various naturalisation variables remain almost identical for both men and women. We therefore conclude that the effect of citizenship is not explained by variation in labour market sectors.

**Table 6:** Individual fixed-effects regression on (CPI-adjusted) Log labour income and (CPI-adjusted) Log labour income when controlling for labour market sectors of male immigrants with paid employment, cohorts 1999–2002. *Source*: Statistics Netherlands

	Men			
	Coef.	SE	Coef.	SE
Naturalisation				
Yes	0.013	0.002***	0.011	0.002***
No	Ref.	Ref.	Ref.	Ref.
Years since migration*naturalisation during observation period				
	0.018	0.000***	0.017	0.000***
Years since naturalisation*naturalisation				
	− 0.017	0.001***	− 0.016	0.001***
Sector				
Agriculture, forestry and fishery			Ref.	Ref.
Non-housing industry and energy			0.067	0.003***
Housing industry			0.067	0.004***
Transportation and communication			− 0.005	0.003
Information and communication			0.021	0.003***
Financial services			0.076	0.004***
Rent and management of property			0.035	0.007***
Business services			0.010	0.003***
Public sector and care sector			0.039	0.003***
Culture, recreation and other			0.031	0.004***
	*N* = 37,114		*N* = 37,114	
	Observations = 408,482		Observations = 408,482	
	− 2 Log-likelihood = 531,423		− 2 Log-likelihood = 527,604	

Results include controls for years since migration, the partner status and having young children in the household

***p < 0.001

**Table 7:** Individual fixed-effects regression on (CPI-adjusted) Log labour income and (CPI-adjusted) Log labour income when controlling for labour market sectors of female immigrants with paid employment, cohorts 1999–2002. *Source*: Statistics Netherlands

	Women			
	Coef.	SE	Coef.	SE
Naturalisation				
Yes	0.007	0.002***	0.006	0.002***
No	Ref.	Ref.	Ref.	Ref.
Years since migration*naturalisation during observation period				
	0.018	0.000***	0.017	0.000***
Years since naturalisation*naturalisation				
	− 0.012	0.001***	− 0.012	0.001***
Sector				
Agriculture, forestry and fishery			Ref.	Ref.
Non-housing industry and energy			0.042	0.004***
Housing industry			− 0.027	0.008***
Transportation and communication			− 0.011	0.004**
Information and communication			0.031	0.004***
Financial services			0.066	0.005***
Rent and management of property			0.004	0.007
Business services			− 0.038	0.004***
Public sector and care sector			0.009	0.004*
Culture, recreation and other			− 0.013	0.004**
	*N* = 31,162		*N* = 31,162	
	Observations = 317,796		Observations = 317,796	
	− 2 Log-likelihood = 420,318		− 2 Log-likelihood = 416,881	

Results include controls for years since migration, the partner status and having young children in the household

*p < 0.05

**p < 0.01

***p < 0.001

## Robustness Analyses

In our analyses, migrants are tracked over time for a maximum period of 10 years. Furthermore, we only analyse migrants who stay long enough to fulfil the residence requirement for naturalisation (and thus are eligible to naturalise at some point during the observation period). However, if migrants emigrate after they become eligible for naturalisation, but before they reach the end of the observation period, they prematurely drop out of the analysis. These right-truncated individuals potentially introduce bias by driving our findings. More specifically, migrants who decide to leave The Netherlands after a relatively short period of residence may do so partly because of negative experiences in the labour market. These unsuccessful migrants are simultaneously unlikely to acquire citizenship. To analyse whether the observed citizenship premium is predominantly driven by these emigrating individuals, we perform the main analysis only for individuals who remain in the dataset for the entire observation period. Findings in Table 13 show that the relevance of naturalisation is stable and does not disappear when right-truncated individuals are removed. We thus conclude that we have no reason to assume that we are overestimating the citizenship premium due to right truncation.

In The Netherlands, information on education is mostly based on survey data and therefore incomplete. For this reason, we refrained from including education in our models. Since the level of education is predominantly stable within individuals during the observation period, the relevance of education is mostly captured by the individual fixed-effects. However, given the importance of education for research on labour market outcomes, we performed a robustness check with the available information on education. Table 14 provides coefficients of employed immigrants for whom the level of education is known, both with and without the inclusion of education to the model. The relevance of naturalisation is comparable to the population in the main model (Table 1, Model 1). In line with our expectations, education is positively associated with income from labour. Note that because of the individual fixed-effects, the effect of education reflects change in the educational level within individuals over the observation period, not variation between individuals. Importantly, the coefficients of naturalisation remain statistically significant when education is included in the model. As such, we have no reason to assume that the relevance of citizenship is explained by differences in education between migrants who naturalise and those who do not.

We observe an increase in earnings leading up to naturalisation and hypothesise that this is due to investments migrants make in anticipation of acquiring citizenship. However, an alternative explanation could be that higher earnings increase the propensity or ability to naturalise. For instance, financial means may provide the opportunity to pay for the costs associated with applying for citizenship and completing the exam. To analyse this reversed causal pathway, we compare migrants who became eligible for citizenship acquisition before and after a restriction in citizenship policy in The Netherlands, namely the introduction of a naturalisation test in 2003. More specifically, we compare migrant cohorts 1996–1997, who could naturalise prior to the policy change, and cohorts 2001–2002, who had to complete the naturalisation test [see Peters et al. (2016, 2018) for a similar approach]. If the effect prior to naturalisation is solely due to increased earnings facilitating naturalisation, then we should observe no differences between the cohorts. But if investment in (language) skills and knowledge of the Dutch society in anticipation of citizenship acquisition also matters, than we would expect a stronger effect under the institutional conditions where these skills are a formal requirement for naturalisation. We can only observe migrants from the early cohort group from 1999 onwards, but these migrants are normally only eligible for naturalisation after this point in time due to the residence requirement of 5 years. Results in Table 15 reveal that the positive labour market outcomes we observe already prior to naturalisation (years since migration*naturalisation during the observation period) are stronger for women during the later cohort group under the more restrictive institutional conditions compared to the earlier cohort group who did not have to successfully complete the naturalisation test. These findings thus suggest that the labour market outcomes prior to naturalisation are not solely due to earnings-specific effects. Also note that the one-time effect of naturalisation is more positive under the more restrictive institutional conditions and actually negative for those under the liberal conditions. Presumably, the signalling effect of the host country citizenship is stronger when access to the status is more exclusive.

To further determine whether the effects prior to naturalisation are due to investment in anticipation of naturalisation, or rather higher earnings increasing the propensity to naturalise, we again estimate the main model with an instrumental variable. We follow the approach of Just and Anderson (2012, p. 499) by using the geographical distance between the host country and the origin country as an instrument for naturalisation. Literature suggests that a shorter distance between the origin and host country makes it easier to maintain ties with the origin country and thus disincentives full integration through naturalisation. In the same vein, more geographical distance increases the costs associated with return migration and thus increases the propensity to naturalise (Yang 1994, p. 473). However, when time-invariant country characteristics such as economic development are held constant through the individual fixed-effects, there is no clear association between geographical distance and earnings from labour. As such, the distance between the host and origin country is a suitable instrument to isolate the association between naturalisation and earnings [see Peters et al. (2018) for a similar approach]. Results in Table 16 show that the coefficient measuring anticipation (years since migration*naturalisation during the observation period) remains positive and statistically significant with our instrumental variable. As an additional robustness check, we use EU/non-EU as an instrument for naturalisation. Originating from an EU country or not is a strong determinant of the propensity to naturalise, yet one could argue that there is no clear association between originating from an EU country or not and earnings from labour when other time-invariant country characteristics are held constant through the individual fixed-effects. Results for the anticipation coefficient are similar to the previous IV-approach. Assuming that these are good instruments for naturalisation,^8^ the findings suggest that the positive labour market outcomes prior to naturalisation are not solely attributable to higher earnings facilitating naturalisation.

## Conclusion

Over the last decades, naturalisation has emerged as a potentially promising vehicle to facilitate the settlement process of immigrants. However, empirical support for a so-called citizenship premium is inconclusive, as some studies reveal a positive effect of naturalisation in terms of income from labour, whereas others do not. In this paper, we theorise on potential explanations for this empirical ambiguity and test these assumptions using register data from Statistics Netherlands.

Our analyses reveal that in general, naturalisation confers a modest one-time boost in immigrant earnings after naturalisation. Although limited evidence for positive signalling is a common observation in the literature (Bratsberg and Raaum 2011, p. 197; Bratsberg et al. 2002, p. 582; Helgertz et al. 2014, p. 352), so far there is no clear explanation why the effect is not more substantial. We argue that the signalling potential of citizenship is particularly relevant to unemployed migrants, since having paid employment potentially serves as a positive signalling device in its own right. Consistent with this argument, we observe a more pronounced positive effect of naturalisation for both men and women when including observations when migrants are unemployed in the model. This suggests that citizenship acquisition particularly facilitates access to the labour market, whereas its effects in terms of occupational mobility are more limited. Given the predominant focus in the literature on immigrant earnings as opposed to employment, this may in part explain the modest empirical support for a citizenship premium in contemporary studies. Furthermore, our findings indicate that citizenship acquisition does not provide an upward shift in earnings for employed migrants from economically more developed countries of origin, whereas we do observe a positive effect among migrants from less developed countries. In other words, insofar as there is a subsequent effect of naturalisation, it matters exclusively to migrants from poorer countries of origin, which is consistent with earlier longitudinal research in the USA (Bratsberg et al. 2002, p. 590). We argue that these migrants face the most structural disadvantages in the labour market and therefore stand to benefit from citizenship most. More detailed analyses show that the relevance of citizenship for employed immigrants in part results from an increase in working hours, but is not explained by variation in labour market sectors.

Consistent with earlier research in Sweden and Denmark (Helgertz et al. 2014, p. 352), the earnings profile of immigrants develops faster prior to naturalisation than afterwards. Detailed analyses confirm that earnings from labour increase leading up to the moment of naturalisation and peak around the moment of citizenship acquisition. We follow the interpretation of Bratsberg et al. (2002, p. 590), who argue that immigrants anticipate naturalisation by investing in their own human capital development. As such, the labour market performance of these migrants already improves prior to naturalisation as a result of the explicit decision to naturalise in the future. This is an important finding for two reasons. First, the observation that the positive effects prior to naturalisation peak around the moment of naturalisation means that scholars who wish to isolate these effects to capture the causal relevance of citizenship need to adjust their model specifications accordingly. Second, understanding citizenship not only as a status but also as a process driven by structured agency emphasises that the pathway to citizenship matters for associated outcomes and may help us theorise on potential explanations for heterogeneous effects of naturalisation which cannot be explained by more traditional mechanisms such as positive signalling. Examples include the relevance of civic and linguistic requirements for naturalisation, as well as the speed of naturalisation. Although the relevance of the institutional context is ideally analysed cross-nationally, our preliminary comparison of migrant cohorts who could naturalise under varying institutional conditions suggest that the citizenship premium is more pronounced under stricter policies. However, the stratifying effect of such requirements can easily turn into mechanisms of exclusion, as restrictive citizenship policies particularly affect the most vulnerable migrant groups who stand to benefit from citizenship most (Peters et al. 2018). Maximising the potential for citizenship to facilitate the integration of immigrants thus constitutes a balancing act. Where exactly this balance lies is a key question for policy makers and a fruitful avenue for future research.

## Appendix

See Tables 8, 9, 10, 11, 12, 13, 14, 15 and 16.

**Table 8:** Descriptive statistics on mean (CPI-adjusted) Log labour income of male and female immigrants with paid employment in percentages, cohorts 1999–2002. *Source*: Statistics Netherlands

	Men			Women		
	Mean	95% conf. interval		Mean	95% conf. interval	
Naturalisation						
No naturalisation	3.2860	3.2849	3.2872	3.1290	3.1276	3.1304
> 3 years prior to naturalisation	3.0730	3.0690	3.0771	2.9468	2.9418	2.9519
3 years prior to naturalisation	3.1060	3.1006	3.1114	2.9762	2.9695	2.9828
2 years prior to naturalisation	3.1378	3.1329	3.1427	3.0128	3.0069	3.0186
1 year prior to naturalisation	3.1611	3.1565	3.1656	3.0345	3.0291	3.0400
Year of naturalisation	3.1739	3.1693	3.1785	3.0536	3.0482	3.0590
1 year after naturalisation	3.1886	3.1838	3.1934	3.0729	3.0675	3.0783
2 years after naturalisation	3.2049	3.1999	3.2099	3.0876	3.0820	3.0932
3 years after naturalisation	3.2070	3.2015	3.2125	3.0938	3.0876	3.1001
> 3 years after naturalisation	3.2203	3.2155	3.2252	3.1054	3.0996	3.1112
Age at migration						
20–24 year	3.1761	3.1743	3.1778	3.0521	3.0501	3.0542
25–29 year	3.2252	3.2237	3.2267	3.1300	3.1280	3.1319
30–34 year	3.2483	3.2463	3.2504	3.1233	3.1206	3.1260
35–39 year	3.2979	3.2949	3.3010	3.0942	3.0905	3.0978
40–44 year	3.3389	3.3344	3.3433	3.0650	3.0602	3.0697
45–50 year	3.3914	3.3852	3.3976	3.0602	3.0535	3.0669
Years since migration						
0–1 years	3.1739	3.1709	3.1769	3.0360	3.0321	3.0400
2–3 years	3.2285	3.2264	3.2306	3.0779	3.0754	3.0804
4–5 years	3.2519	3.2499	3.2539	3.0975	3.0952	3.0998
6–7 years	3.2635	3.2616	3.2655	3.1136	3.1113	3.1158
8–9 years	3.2676	3.2656	3.2696	3.1222	3.1198	3.1246
Partner						
No partner	3.2065	3.2047	3.2082	3.1690	3.1666	3.1713
Foreign-born foreign partner	3.3069	3.3051	3.3087	3.1007	3.0984	3.1029
Foreign-born Dutch partner	3.1639	3.1620	3.1658	2.9202	2.9171	2.9233
Native-born Dutch partner	3.2694	3.2672	3.2715	3.1080	3.1062	3.1098
Children < 18 in household						
Yes	3.2639	3.2624	3.2654	3.0218	3.0200	3.0236
No	3.2273	3.2260	3.2285	3.1565	3.1550	3.1579
Development country of origin						
Lowest quartile	3.1149	3.1131	3.1168	2.9748	2.9725	2.9771
Second quartile	3.1643	3.1626	3.1660	3.0182	3.0161	3.0203
Third quartile	3.2611	3.2595	3.2628	3.1479	3.1458	3.1501
Highest quartile	3.4463	3.4444	3.4482	3.2563	3.2541	3.2584
EU country of origin						
Yes	3.3827	3.3811	3.3843	3.2136	3.2120	3.2153
No	3.1857	3.1846	3.1868	3.0296	3.0282	3.0311
Total	3.2432	3.2423	3.2442	3.0967	3.0955	3.0978
	*N* = 40,204			*N* = 34,327		
	Observations = 502,962			Observations = 389,668

**Table 9:** Descriptive statistics on background characteristics of migrants with paid employment who (do not) naturalise within the observation period, cohorts 1999–2002. *Source*: Statistics Netherlands

	Naturalisation during observation period		No naturalisation during observation period	
	Obs.	%	Obs.	%
Gender				
Male	158,589	54.5	344,373	57.3
Female	132,626	45.5	257,042	42.7
Age at migration				
20–24 year	79,324	27.2	140,326	23.3
25–29 year	102,732	35.3	181,881	30.2
30–34 year	60,814	20.9	126,958	21.1
35–39 year	28,795	9.9	78,367	13.0
40–44 year	13,840	4.8	46,222	7.7
45–50 year	5710	2.0	27,661	4.6
Years since migration				
0–1 years	23,725	8.1	80,404	13.4
2–3 years	52,142	17.9	129,619	21.6
4–5 years	64,745	22.2	137,953	22.9
6–7 years	75,644	26.0	133,170	22.1
8–9 years	74,959	25.7	120,269	20.0
Partner				
No partner	64,083	22.0	178,713	29.7
Foreign-born foreign partner	75,813	26.0	198,811	33.1
Foreign-born Dutch partner	68,925	23.7	69,054	11.5
Native-born Dutch partner	82,394	28.3	154,837	25.7
Children < 18 in household				
Yes	147,970	50.8	244,255	40.6
No	143,245	49.2	357,160	59.4
Development country of origin				
Lowest quartile	114,717	39.4	105,630	17.6
Second quartile	100,220	34.4	132,482	22.0
Third quartile	66,121	22.7	156,203	26.0
Highest quartile	10,157	3.5	207,100	34.4
EU country of origin				
Yes	16,469	5.7	272,323	45.3
No	274,746	94.3	329,092	54.7
Total	291,215	100.0	601,415	100.0
	*N* = 26,656		*N* = 47,875	
	Observations = 291,215		Observations = 601,415

**Table 10:** Individual fixed-effects regression on (CPI-adjusted) Log labour income of male and female immigrants with paid employment, by development country of origin, cohorts 1999–2002. *Source*: Statistics Netherlands

	Men				Women				
	Low development		High development		Low development			High development	
	Coef.	SE	Coef.	SE	Coef.	SE		Coef.	SE
Naturalisation									
Yes	0.014	0.002***	0.007	0.004	0.020	0.003***		− 0.002	0.005
No	Ref.	Ref.	Ref.	Ref.	Ref.	Ref.		Ref.	Ref.
Years since migration*naturalisation during observation period									
	0.018	0.001***	0.021	0.001***	0.015	0.001***		0.017	0.001***
Years since naturalisation*naturalisation									
	− 0.018	0.001***	− 0.020	0.001***	− 0.012	0.001***		− 0.017	0.002***
	*N* = 24,848		*N* = 22,907		*N* = 21,480			*N* = 16,755	
	Observations = 257,952		Observations = 245,010		Observations = 203,750			Observations = 185,918	
	− 2 Log-likelihood = 525,244		− 2 Log-likelihood = 399,953		− 2 Log-likelihood = 384,844			− 2 Log-likelihood = 324,811	

Results include controls for years since migration, the partner status and having young children in the household

***p* < 0.01

****p* < 0.001

**Table 11:** Percentages labour market sector by naturalisation of male and female immigrants with paid employment, cohorts 1999–2002. *Source*: Statistics Netherlands

	Men		Women	
	Not naturalised	Naturalised	Not naturalised	Naturalised
*Sector*				
Agriculture, forestry and fishery	2.1	1.6	2.0	1.1
Non-housing industry and energy	14.8	17.2	7.7	6.2
Housing industry	3.1	3.3	0.4	0.7
Transportation and communication	26.6	30.9	23.5	22.8
Information and communication	5.5	2.6	3.9	2.7
Financial services	2.1	1.4	2.7	3.4
Rent and management of property	0.3	0.4	0.4	0.3
Business services	35.9	30.0	36.8	30.9
Public sector and care sector	7.2	10.3	18.8	28.5
Culture, recreation and other	2.4	2.4	3.8	3.4
Total	100.0	100.0	100.0	100.0
	*N* = 37,114		*N* = 31,162	
	Observations = 408,482		Observations = 317,796

**Table 12:** Descriptive statistics on mean (CPI-adjusted) Log labour income per sector of male and female immigrants with paid employment, cohorts 1999–2002. *Source*: Statistics Netherlands

	Men			Women		
	Mean	95% conf. interval		Mean	95% conf. interval	
*Sector*						
Agriculture, forestry and fishery	3.1501	3.0909	3.2093	3.0198	2.9666	3.0730
Non-housing industry and energy	3.3261	3.2835	3.3687	3.2521	3.2007	3.3035
Housing industry	3.2979	3.2639	3.3319	3.0936	3.0239	3.1633
Transportation and communication	3.2102	3.1439	3.2765	3.1184	3.0569	3.1799
Information and communication	3.4660	3.4052	3.5268	3.3716	3.3212	3.4220
Financial services	3.6167	3.5517	3.6817	3.4270	3.3717	3.4823
Rent and management of property	3.2617	3.1984	3.3250	3.1347	3.0712	3.1982
Business services	3.2374	3.1755	3.2993	3.0374	2.9431	3.1317
Public sector and care sector	3.3170	3.2712	3.3628	3.1626	3.1107	3.2145
Culture, recreation and other	3.2521	3.1876	3.3166	3.0871	3.0225	3.1517
Total	3.2694	3.2084	3.3305	3.1234	3.0595	3.1873
	*N* = 37,114			*N* = 31,162		
	Observations = 408,482			Observations = 317,796

**Table 13:** Individual fixed-effects regression on (CPI-adjusted) Log labour income of male and female immigrants with and without paid employment, no right truncation *Source*: Statistics Netherlands

	Model 1				Model 2			
	Men		Women		Men		Women	
	Coef.	SE	Coef.	SE	Coef.	SE	Coef.	SE
Naturalisation								
Yes	0.011	0.002***	0.009	0.002***	0.117	0.009***	0.119	0.009***
No	Ref.	Ref.	Ref.	Ref.	Ref.	Ref.	Ref.	Ref.
Years since migration*naturalisation during observation period								
	0.020	0.001***	0.020	0.001***	0.143	0.002***	0.120	0.002***
Years since naturalisation*naturalisation								
	− 0.017	0.001***	− 0.012	0.001***	− 0.107	0.003***	− 0.079	0.003***
	*N* = 34,506		*N* = 30,148		*N* = 34,506		*N* = 30,148	
	Observations = 440,444		Observations = 347,800		Observations = 675,315		Observations = 598,201	
	− 2 Log-likelihood = 87,061		− 2 Log-likelihood = 90,940		− 2 Log-likelihood = 1,965,778		− 2 Log-likelihood = 1,876,742	

Results include controls for years since migration, the partner status and having young children in the household

Model 1 is restricted to observations of individuals with paid employment. Model 2 includes observations in which individuals are unemployed

Cohorts 1999–2002

****p* < 0.001

**Table 14:** Individual fixed-effects regression on (CPI-adjusted) Log labour income of male and female immigrants with paid employment, cohorts 1999–2002. *Source*: Statistics Netherlands

	Men		Women		Men		Women	
	Coef.	SE	Coef.	SE	Coef.	SE	Coef.	SE
Naturalisation								
Yes	0.016	0.003***	0.024	0.003***	0.011	0.003***	0.015	0.003***
No	Ref.	Ref.	Ref.	Ref.	Ref.	Ref.	Ref.	Ref.
Years since migration*naturalisation during observation period								
	0.023	0.001***	0.024	0.001***	0.021	0.001***	0.021	0.001***
Years since naturalisation*naturalisation								
	− 0.012	0.001***	− 0.015	0.001***	− 0.011	0.001***	− 0.013	0.001***
Education								
Low					Ref.	Ref.	Ref.	Ref.
Middle					0.038	0.003***	0.060	0.003***
High					0.144	0.004***	0.193	0.004***
	*N* = 17,337		*N* = 15,936		*N* = 17,337		*N* = 15,936	
	Observations = 119,911		Observations = 108,582		Observations = 119,911		Observations = 108,582	
	− 2 Log-likelihood = 180,104		− 2 Log-likelihood = 163,149		− 2 Log-likelihood = 178,879		− 2 Log-likelihood = 161,093	

Results include controls for years since migration, the partner status and having young children in the household

****p* < 0.001

**Table 15:** Individual fixed-effects regression on (CPI-adjusted) Log labour income of male and female immigrants with paid employment, cohorts 1996–1997 and 2001–2002. *Source*: Statistics Netherlands

	Cohort 1996–1997				Cohort 2001–2002			
	Men		Women		Men		Women	
	Coef.	SE	Coef.	SE	Coef.	SE	Coef.	SE
Naturalisation								
Yes	− 0.014	0.002***	− 0.007	0.003**	0.020	0.003***	0.014	0.003***
No	Ref.	Ref.	Ref.	Ref.	Ref.	Ref.	Ref.	Ref.
Years since migration*naturalisation during observation period								
	0.019	0.001***	0.013	0.001***	0.018	0.001***	0.020	0.001***
Years since naturalisation*naturalisation								
	− 0.019	0.001***	− 0.013	0.001***	− 0.013	0.001***	− 0.010	0.001***
	*N* = 22,607		*N* = 19,081		*N* = 20,874		*N* = 18,137	
	Observations = 242,571		Observations = 178,674		Observations = 258,337		Observations = 202,707	
	− 2 Log-likelihood = 472,839		− 2 Log-likelihood = 323,443		− 2 Log-likelihood = 818,947		− 2 Log-likelihood = 576,230	

Results include controls for years since migration, the partner status and having young children in the household

***p* < 0.01

****p* < 0.001

**Table 16:** Individual fixed-effects regression on (CPI-adjusted) Log labour income of male and female immigrants with paid employment, cohorts 1999–2002. *Source*: Statistics Netherlands

	Men		Women		Men		Women		
	Coef.	SE	Coef.	SE	Coef.	SE	Coef.	SE	
Naturalisation									
Yes	0.035	0.002***	0.034	0.002***	0.030	0.002***	0.027	0.002***	
No	Ref.	Ref.	Ref.	Ref.	Ref.	Ref.	Ref.	Ref.	
Years since migration*distance origin and host country									
	0.005	0.000***	0.005	0.000***					
Years since migration*EU origin country									
					0.008	0.000***	0.010	0.000***	
Years since naturalisation*naturalisation									
	− 0.004	0.001***	0.002	0.001**	− 0.005	0.001***	− 0.001	0.001	
	*N* = 40,204		*N* = 34,327		*N* = 40,204		*N* = 34,327		
	Observations = 502,962		Observations = 389,668		Observations = 502,962		Observations = 389,668		
	− 2 Log-likelihood = 948,673		− 2 Log-likelihood = 721,571		− 2 Log-likelihood = 948,180		− 2 Log-likelihood = 721.180		

Results include controls for years since migration, the partner status and having young children in the household

***p* < 0.01

****p* < 0.001

## Footnotes

1 Examples include Bevelander and Veenman (2008), Devoretz and Pivnenko (2006), Enchautegui and Giannarelli (2015), Fougère and Safi (2009), Scott (2008) and Sumption and Flamm (2012).

2 See Bratsberg and Raaum (2011, p. 198), Engdahl (2014, p. 17) and Peters et al. (2018, pp. 1055–1057) for similar arguments.

3 We performed the same analyses including migrants who do not stay long enough to become eligible for naturalisation. Results were highly similar to those reported in the paper.

4 Note that the mean number of observations per individual is 12.5 and 11.4 for men and women, respectively (out of a maximum of 20). The reason for this is twofold. First, Table 8 focuses on observations in which migrants are employed and ignores observations where migrants are unemployed. Specific analysis in this paper includes observations where migrants are unemployed (Table 1, Model 2 and Table 13, Model 2). Second, Table 8 does not account for outmigration. When controlling for outmigration, the mean number of observations per individual increases substantially to 19.6 and 19.8 for men and women, respectively (see Table 13, Model 2). It is not exactly 20 due to a very small number of instances where information on the economic development or political stability of origin countries was unknown for specific years.

5 See also Table 9 in the Appendix for descriptive statistics on naturalization by individual and origin country characteristics.

6 Note that this needs to be considered with care in light of potential floor effects, since unemployed migrants have value zero on the dependent variable. More generally, an analysis focusing on income from labour is not ideally suited to assess the relevance of citizenship for employment. However, a similar study in the Netherlands using logistic individual fixed-effects regression also reveals a substantial positive effect of naturalisation on employment compared to the literature focusing on wages (Peters et al. 2018). We thus are confident that the one-time boost after naturalisation is indeed stronger for employment rather than earnings, but emphasize that our findings in Table 1, Model 2 are principally meant to highlight our theoretical argument, rather than determinately establishing this point empirically.

7 Subsequent analyses again focus on models based on Eq. (1). However, distributed individual fixed-effects regressions have been performed for all models as well. The observed patterns are similar in all analyses, namely an increase in Log labour income leading up to naturalisation, which flattens out after the host country citizenship is acquired.

8 The first-stage F-statistic is > 10 in both models (Staiger and Stock 1997).
